# Position statement and updated international guideline for safe and effective whole-body electromyostimulation training-the need for common sense in WB-EMS application

**DOI:** 10.3389/fphys.2023.1174103

**Published:** 2023-03-22

**Authors:** Wolfgang Kemmler, Michael Fröhlich, Oliver Ludwig, Christoph Eifler, Simon von Stengel, Sebastian Willert, Marc Teschler, Anja Weissenfels, Heinz Kleinöder, Florian Micke, Nicolas Wirtz, Christoph Zinner, Andre Filipovic, Bernd Wegener, Joshua Berger, Alexandre Evangelista, Stefano D’Ottavio, Jaskanwal Deep Singh Sara, Amir Lerman, Unai A. Perez de Arrilucea Le Floc’h, Abraham Carle-Calo, Angel Guitierrez, Francisco J. Amaro-Gahete

**Affiliations:** ^1^ Institute of Radiology, University Hospital Erlangen (UKER), Erlangen, Germany; ^2^ Department of Sports Science, Rheinland-Pfälzische Technische Universität Kaiserslautern-Landau, Kaiserslautern, Germany; ^3^ German University for Prevention and Health Management, Saarbrücken, Germany; ^4^ Department of Rehabilitation Sciences, University of Witten/Herdecke, Witten, Germany; ^5^ Institute of Sports Science, Friedrich-Alexander University of Erlangen-Nürnberg, Erlangen, Germany; ^6^ Institute of Training Science and Sport Informatics, German Sport University Cologne, Cologne, Germany; ^7^ Central Library for Sport Sciences, German Sport University Cologne, Cologne, Germany; ^8^ University of Applied Sciences for Police and Administration of Hesse, Wiesbaden, Germany; ^9^ Soccer Club Paderborn 07, Paderborn, Germany; ^10^ Musculoskeletal University Center, Ludwig-Maximilian-University of Munich, Munich, Germany; ^11^ Experimental Physiology and Biochemistry, Center for Physical Education and Sport, University Federal do Espírito Santo, Vitória, Brazil; ^12^ Clinical Sciences and Translational Medicine Department, University of Rome, Rome, Italy; ^13^ Department of Cardiovascular Medicine, Mayo College of Medicine, Rochester, MN, United States; ^14^ Department of Physical Education and Sport, University of Granada, Granada, Spain; ^15^ Department of Physiology, University of Granada, Granada, Spain; ^16^ Centro de Investigación Biomédica en Red Fisiopatología de la Obesidad y Nutrición (CIBERobn), Instituto de Salud Carlos III, Madrid, Spain; ^17^ Instituto de Investigación Biosanitaria de Granada (ibs.GRANADA), Granada, Spain

**Keywords:** recommendations, international consensus, whole-body electromyostimulation, electrical muscle stimulation, safety

## Abstract

Whole-Body Electromyostimulation (WB-EMS) is a training technology that enables simultaneous stimulation of all the main muscle groups with a specific impulse intensity for each electrode. The corresponding time-efficiency and joint-friendliness of WB-EMS may be particularly attractive for people unable or unmotivated to conduct (intense) conventional training protocols. However, due to the enormous metabolic and musculoskeletal impact of WB-EMS, particular attention must be paid to the application of this technology. In the past, several scientific and newspaper articles reported severe adverse effects of WB-EMS. To increase the safety of commercial non-medical WB-EMS application, recommendations “for safe and effective whole-body electromyostimulation” were launched in 2016. However, new developments and trends require an update of these recommendations to incorporate more international expertise with demonstrated experience in the application of WB-EMS. The new version of these consensus-based recommendations has been structured into 1) “general aspects of WB-EMS”, 2) “preparation for training”, recommendations for the 3) “WB-EMS application” itself and 4) “safety aspects during and after training”. Key topics particularly addressed are 1) consistent and close supervision of WB-EMS application, 2) mandatory qualification of WB-EMS trainers, 3) anamnesis and corresponding consideration of contraindications prior to WB-EMS, 4) the participant’s proper preparation for the session, 5) careful preparation of the WB-EMS novice, 6) appropriate regeneration periods between WB-EMS sessions and 7) continuous interaction between trainer and participant at a close physical distance. In summary, we are convinced that the present guideline will contribute to greater safety and effectiveness in the area of non-medical commercial WB-EMS application.

## 1 Objectives and rationale for the recommendations for safe and effective WB-EMS

Considering its ability to affect large muscle groups in parallel and with dedicated intensity, whole-body electromyostimulation (WB-EMS) has been positioned as a very time efficient training option for improving health and performance in humans (Kemmler et al., 2016b; Kemmler et al., 2016c). However, this unique simultaneous stimulation of all the main muscle groups—sometimes in excess with supra-maximum intensity—entails potential risks, thus requiring a responsible approach to this training technology and its application. After the publication of some case studies (Kastner et al., 2014; Finsterer and Stollberger, 2015; Malnick et al., 2016) and public media releases that reported negative side effects (in particular severe rhabdomyolysis) predominately after an initial WB-EMS application (Habich, 2015) the first calls for official regulation of WB-EMS were published in 2016 (Malnick et al., 2016). More reliable data on negative side effects of inadequate WB-EMS application (Teschler et al., 2016), the early launch (i.e., in about 2007) and the impressive number of some 2,500 commercial WB-EMS facilities in Germany might explain the concerted efforts of national WB-EMS research groups to ensure safe and effective WB-EMS application through dedicated recommendations in 2016 (Kemmler et al., 2016a). Although this “Guideline for safe and effective whole-body electromyostimulation” was not a mandatory norm or even a dedicated clinical practice guideline, we were convinced that the voluntary implementation of the recommendations by most providers has contributed to more safety and effectiveness in the area of non-medical commercial WB-EMS application. However, new trends in WB-EMS settings, (e.g., non- or semi-supervised groups, individual or group remote WB-EMS at home or flat-rate WB-EMS) have once again challenged safety and effectiveness aspect of WB-EMS application. This led us to update the 2016 recommendation for WB-EMS at an international level in the light of the growing international market of WB-EMS. However, the present “guideline for safe and effective whole-body electromyostimulation training” that focuses on the application of an exercise technology cannot been compared with the much more elaborate and extensive clinical practice guidelines that focus on conditions or diseases in dedicated cohorts. Instead, the present work should be considered as evidence-based expert recommendations and guidance for users. In the present article that focuses on commercial non-medical WB-EMS, we have summarized our recommendations for safe and effective WB-EMS application in adult humans.

## 2 Methods

We conducted a simplified multi-step Delphi method (Verhagen et al., 1998) to achieve expert consensus: 1) Forming an expert panel, 2) individual revision of the 2016 recommendations (Kemmler et al., 2016a) by all working groups considering critical aspects raised and submitted by the study coordinator (UKER, Germany), 3) Structured anonymized rounds that focus on revisions and specifications of the drafts to establish consensus, and 4) obtaining selected stakeholder input and finalizing the recommendations.

1) We invited the most productive and relevant research groups on WB-EMS (Rodrigues-Santana et al., 2022) so as to generate a broad base for consensus-driven recommendations. In detail, scientific expert groups from Brazil (AE), Germany (MF, OL, CE, MT,AW, HK, FM, NW, CZ, AF, BW, and JB), Italy (SD), Spain (UP, ACC, AG, and FAG) and the US (JD and AL) responded to the invitation of the UKER, Germany (WK, SvS, and SB) that initiated and coordinated the consensus process. 2) All expert groups were provided with the Microsoft Word version of the 2016 recommendation (Kemmler et al., 2016a) that was revised by the coordinating institution (UKER) using the word track changes and comment function to highlight critical aspects. Expert groups were asked to comment on these positions but were also free to address additional critical aspects which in their opinion ought to be discussed. 3) Revisions and aspects addressed by the expert groups were collected and considered by WK and SvS (UKER, Germany) to identify areas of agreement and disagreement. Of importance, there was no fundamental disagreement between the groups, however, in some cases recommendations were specified or extended during the second round of the process. Including and highlighting the revisions in the manuscript (WK and SvS), all groups were provided with the new draft, without information about the source of the revision. All expert groups agreed with the revised recommendations, thus the process was completed after the second round. 4) Teaching institutions (*n* = 3) and selected commercial facilities (*n* = 3), all members of the “WB-EMS round table Germany,” were asked to review and comment on utility and clarity of the final draft. After corresponding feedback and minor revisions, all expert groups and stakeholders approved the final draft of the present “expert guideline”.

## 3 Explanations and considerations of the recommendations

Of note, this consortium does not always agree on everything, this is particularly the case concerning aspects of supervision and training frequency of WB-EMS application. Indeed, while some aspects are debatable, the strategy of prioritizing safety aspects (“safety first”), potentially at the expense of maximum effectiveness (at least in non-athletic cohorts), has been finally accepted by all the research groups involved. As some points in the guideline might not be immediately self-explanatory for the reader, we would like to clarify some particularly crucial and debatable recommendations below. Supervision by qualified trainers of the WB-EMS application has been given high priority in this guideline. In contrast to other types of exercise (e.g., dynamic resistance exercise), endogenous protective mechanisms do not reliably prevent overload caused by the exogenous electrical stimulation. Furthermore, the whole-body approach with at least six electrodes (Kemmler et al., 2020) raises the problem of generating adequate stimulus intensity per electrode consistently during the session. We conclude that these features must be necessarily addressed through close supervision by experienced trainers who constantly visually control and frequently request participant feedback related to perceived exertion and adequate intensity per electrode. Additionally, a close physical distance between trainer and participant is important for maintaining eye contact, for haptically correcting the trainee and for immediately stopping the application in case of emergency or unintended side effects. Thus, the physical presence of the trainer during all training sessions is indispensable. Apart from the “safety” aspect, it is noticeable that all reliable published clinical studies which reported positive effects of WB-EMS training on both sports performance and human health applied a low supervision ratio (Kemmler et al., 2021; Micke et al., 2022). This feature indicates the crucial role of consistent and close supervision for the management of adequate impulse intensity specification as a key factor of successful WB-EMS application. In summary, we thus strictly advise against un- or remotely-supervised application of WB-EMS without the guidance of a licensed trainer, not only to prevent unintended side effects and hazards, but also to ensure effective WB-EMS application.

The most severe side effects reported after the application of WB-EMS (e.g., severe exertional rhabdomyolysis) occurred in WB-EMS novices—largely independently of their general training status [e.g., (Kastner et al., 2014; Finsterer and Stollberger, 2015; Hong et al., 2016; Teschler et al., 2016; Hettchen et al., 2019; Johannsen and Krogh, 2019)]. As much as a 1000-fold (240.000 U/l) increase in creatine-kinase (CK) baseline levels was reported for a young professional soccer player after a (too) intensive initial WB-EMS application (Kastner et al., 2014). These results were confirmed by Teschler et al. (2016) who applied (very) high intensity WB-EMS under close medical supervision in a cohort of 26 healthy young volunteers without prior WB-EMS experience (Teschler et al., 2016). Observing high heterogeneity between the individuals peak CK-levels (range: 2366–143674 IU/l), another important finding was the delayed CK-peak observed 72 h post exercise. The latter results led us to recommend the limitation of once weekly WB-EMS application during the conditioning phase, even when applying lower impulse intensity to prevent excessive muscular damage and corresponding accumulation of biomarkers so as to protect individuals particularly sensitive to WB-EMS. After 10 weeks of once weekly WB-EMS training however, an identically (very) high intensity WB-EMS session (Teschler et al., 2016) resulted in moderate increases of CK (335–1987 IU/l) and myoglobin (137–712 μg/l) concentrations 72 h post exercise, i.e., levels comparable to the results observed after (eccentric) resistance exercise (Koch et al., 2014).

There is some evidence that the pronounced repeated bout effect (Brown et al., 1997; Nosaka et al., 2011) on WB-EMS induced CK and myoglobin increases started earlier (Hettchen et al., 2019). Nevertheless, current research suggests a period of approximately 1 month to ensure optimal muscle cell regeneration after the first time the muscle is exposed to severe EMS-induced muscle damage (Mackey and Kjaer, 2017a; Mackey and Kjaer, 2017b). Although initial WB-EMS application should not result in muscle damage, we recommend an eight-to 10-week familiarization and conditioning period with decreased impulse intensity-intensity, duration and exercise frequency.

Another recommendation derived from the findings of Teschler et al. (2016) refers to the 4-day recovery period between high intensity sessions after a 10-week conditioning phase. Although a considerable body of evidence recommends a training frequency of 1.5 sessions/week for obtaining significant positive effects on various outcomes (particularly in non-athletic cohorts), some additional aspects should be addressed. First, there is a relative lack of long-term (≥12 months) WB-EMS trials (von Stengel et al., 2015). Thus, the evidence of attenuating long-term effects of unaltered low-training frequency cannot be ruled out, despite maintaining the stimulus intensity at supra-threshold level due to the individual RPE specification. Future long-term studies should address this important issue whilst also assessing potential long-term adverse effects of WB-EMS. However, from a pragmatic point of view, more than 15 years of commercial WB-EMS application with thousands of long-term users should have provided evidence for serious adverse effects of long-term WB-EMS application. Another argument for a low (training) frequency of WB-EMS is the resistance type (RT) character of present WB-EMS applications. Although WB-EMS can be also applied as a high frequency - low intensity setting, i.e., an endurance type exercise, the minor additional effect of WB-EMS superimposed on running or cycling [e.g., (Mathes et al., 2017; Amaro-Gahete et al., 2018; Filipovic et al., 2019)] and the loss of the time effective character (Kemmler et al., 2021), speak against such a protocol.

Finally and with respect to training frequency in athletic cohorts, the WB-EMS application strategy considerably varies from conventional non-medical application. “Superimposed WB-EMS” (e.g., exercises including jumps or short sprints) with high voluntary effort supported by moderate impulse-intensity EMS is the method of choice in athletic populations (Micke et al., 2022). Considering 1) The lower impulse intensity used, 2) the more sophisticated regeneration methods and 3) the medical supervision and support may justify a slightly higher training frequency in athletes than the recommended by the present guideline. Furthermore, there is some evidence that, from a physiological point of view, athletes show better neuromuscular adaptation to external load stimuli than non-trained individuals (Seyri and Maffiuletti, 2019).

A crucial issue of WB-EMS application not addressed here is the absolute and relative contra-indications for commercial, non-medical WB-EMS. (Kemmler et al., 2019). Reviewing the present contra-indications in detail, it can be thought that some of the diseases included in the list of absolute contra-indications (e.g., Diabetes Mellitus (van Buuren et al., 2015) or Cancer (Schink et al., 2018) could be safely addressed by WB-EMS after careful medical anamnesis, competent and close supervision by an expert trainer, and the ensuring of rapid medical care in an emergency. We would agree with this position; on the other hand, however and despite the mandatory WB-EMS qualification, e.g., in Germany (BMU, 2020), we doubt that the present trainer qualifications reliably enable the safe handling of high-risk patients. Therefore, EMS for participants with contraindications we currently position WB- in the area of medical EMS, until further studies involving the medical population are available for recommendation”.

Nevertheless, some of the absolute contraindications are debatable and could be changed into relative contraindications or even removed in future recommendations. This process should include more evidence-based WB-EMS research. However, we encourage a (more) profound education of trainers and others (e.g., EMS studio staff) involved in the application of WB-EMS in participants at increased risk for adverse effects. This particularly includes trainer being enabled to detect and differentiate between different motor thresholds based on the visual perception of the muscle contraction and joint movement as well as the time under tension (Alon et al., 1987; Herrero et al., 2006; Maffiuletti et al., 2008).

## 4 Guideline for safe and effective whole-body electromyostimulation

### 4.1 Definitions

#### 4.1.1 Whole-body electromyostimulation (WB-EMS)

“Simultaneous application of electric stimuli *via* at least six current channels or participation of all major muscle groups, with a current impulse effective to trigger muscular adaptations” (Kemmler et al., 2020).

#### 4.1.2 Medical WB-EMS

Medical WB-EMS training is a 1) primarily therapeutic intervention 2) based on an existing diagnosis 3) that is provided by qualified medical–therapeutic personnel 4) in compliance with current guidelines and 5) using medical devices. (Berger et al., 2022).

### 4.2 In general

1. As with other types of exercise training performed at high intensity, it may well be advisable to have a sports medical examination prior to the WB-EMS training.
2. In order to be safe and effective, WB-EMS training must be provided and supervised by a licensed and ideally experienced WB-EMS trainer or, in a university or clinical setting by scientifically trained staff familiar with high knowledge of its application. Non-supervised WB-EMS application must be strictly avoided.
3. Trainers must have official basic education that qualifies them as coaches according to the laws of their country. In addition to a basic exercise and medical qualification, the licensing process of the trainer should include at least 20 h training on dedicated WB-EMS theory and a practical part provided by an accredited educational qualified institution (BMU, 2020).
4. We strongly advise a 1:1 trainer-participant ratio (Medical WB-EMS), although a 1:2 ratio is also considered acceptable for non-medical WB-EMS applications with less critical participants.
5. Prior to the first WB-EMS session, a detailed anamnesis of possible absolute and relative contraindications (Deutsches Institut für Normung, 2019; Kemmler et al., 2019)—based on a list of questions must be performed and documented, confirmed by the client’s signature and archived. While absolute contraindications prevent WB-EMS application in a non-medical WB-EMS setting, a medical practitioner has to give written approval for WB-EMS-application in cases of relative contraindications.
6. In parallel, after detailed personal information on WB-EMS application an informed consent contract should be signed by the clients/participants to ensure the user understands all risks and features of WB-EMS application. We strongly recommend repeating this process at least every 6 months to update changes in client’s health, needs or requests that trainers and other responsible should take into consideration.

### 4.3 Preparing for training

1. As with any kind of intensive exercise training, WB-EMS training should be only carried out in a proper physical condition and state. This includes abstaining from alcohol consumption, drugs, muscle relaxants or severe stress sufficiently long before (i.e., 24–48 h) the training. WB-EMS is also prohibited when suffering from an illness with fever.
2. WB-EMS training can generate very high metabolic stress because of its derived simultaneous stimulation of all the main muscle groups with high intensity (see below). To prevent weakness, dizziness or other adverse effects related to hypoglycemia during the WB-EMS session, sufficient food intake predominately based on carbohydrates should be ensured in preparation of the session. At least a high carbohydrate, but light snack (≈250 kcal) is recommended, ideally 2 h before the WB-EMS training.
3. In parallel, to minimize renal stress related to intense WB-EMS training, which might be particularly important for individuals with undiagnosed renal problems, additional fluids should be scheduled 30 min before and immediately after training (each 250–500 ml or 5 ml/kg body-mass).
4. The trainer has to check the aforementioned aspects before the start of the WB-EMS application by visual inspection and oral inquiry. Electrode location and proper suit adjustment have to be checked prior the start of the WB-EMS session.

### 4.4 During the training

1. Regardless of the health and exercise status or the participant’s ideas and preferences, initial WB-EMS application(s) must be applied carefully. This rules out in particular WB-EMS with high intensity, let alone to exhaustion, during the first 8–10 weeks of WB-EMS application (Teschler et al., 2016).
2. After initial moderate-intensity WB-EMS, i.e., “4” (= somewhat strong) on the Borg CR10 (Borg and Borg, 2010) RPE scale, the stimulation level or impulse intensity can be subsequently increased and adapted to the individual training aims during the next 7–9 weeks. Intensity levels of “7–8” (=very hard) on Borg CR10 (Borg and Borg, 2010) can be allowed after 8–10 sessions of regular training at the earliest. Training to complete exhaustion (”10” at Borg CR10) or continuous tetanus during the impulse phase must be strictly avoided independent of the training status of the individual.
3. Due to individual differences in impulse sensitivity and tolerance, we recommend using the CR10 RPE-scale of Borg (Borg and Borg, 2010) to prescribe, query and monitor the impulse intensity during the WB-EMS session. Of importance, impulse intensity has to be specified, queried and monitored for several times during the entire session for each individual electrode.
4. Body awareness, individual evaluation and interpretation of the perceived exertion should be a focus of the first exercise sessions.
5. In addition to impulse-intensity issues, the first WB-EMS session should be conducted with a reduced volume. We advise 1) 5 min impulse familiarization using a continuous WB-EMS protocol and 2) 12 min of intermittent WB-EMS with short impulse phases (≈4 s), and slow-moderate impulse increases (≈0.3–0.5 s ramps), intermitted by short breaks ((≈4 s). After 4–6 weeks of familiarization/conditioning the WB-EMS session can be carefully increased to a maximum of 20 min applying an intermittent resistance exercise training (RT)-type protocol with high impulse intensities (see above) or 30–40 min session applying an endurance type protocol that was further recommended to consistently schedule (only) moderate impulse intensity (5-6, i.e., “hard” to “hard+” on Borg CR10).
6 Further, for adequate recovery and adaptation and to prevent potential health impairments, the training frequency may not exceed one training session of 20 min per week during the initial 8–10 weeks.
7 After this 8–10-week familiarization and conditioning period, there must be at least a 4-day pause between intense WB-EMS sessions (≥7 Borg CR10) to avoid accumulation of muscle breakdown products permitting adequate regeneration and adaptation.

### 4.5 Safety aspects during and after training

1. During the WB-EMS session, the trainer has to exclusively focus on the wellbeing of the clients/participants. Before, during and after the training session, the trainer has to verbally and visually check the participant’s condition to rule out health risks and ensure effective training. The training session has to be stopped immediately in case of any adverse effects or problems observed by the trainer or raised by the participants.
2. We strongly recommend a very close interaction and proximity between trainer and participant and maintaining a specific focus on the following key points: 1) Frequent feedback about perceived exertion for each area of stimulation, 2) permanent visual monitoring of the participant and eye contact to check participant strain, avoid overload and to react immediately to the first signs of adverse effects, and 3) verbal and haptic movement corrections and rapid assistance in cases of emergency.
3. With respect to the frequent request for higher impulse intensity mentioned above, we suggest checking the adequacy of impulse intensity at least 3 times per area/electrode by verbal query. In cases of inadequate impulse intensity, levels should be adjusted in close interaction between trainer and participants to ensure a safe and effective WB-EMS application.
4. Operating controls must be constantly in reach of the trainer and the participant (i.e., maximum distance of 120 cm [Deutsches Institut für Normung, 2020)] in order to stop the WB-EMS application immediately in case of emergency. The participant has to be briefed on the emergency shutdown function of the device.
5. Medical consultation and clarification is advisable in the case of relevant discomfort, cardiometabolic difficulties or orthopedic problems potentially related to the WB-EMS application. This also refers to hematuria (e.g., cola-colored urine), persistent headache and inflammatory or bleeding problems after potentially too intense WB-EMS application.

## 5 Summary

Based on its ability to simultaneous stimulate all of the main muscle groups with, in excess supra-maximum, (impulse) intensity, WB-EMS is an effective, albeit potentially harmful technology when applied wrongly. A competent and responsible WB-EMS application is crucial to generate positive effects but to avoid adverse effects. The present recommendations should be considered as a guidance for trainers and applicants to realize this aim.

## Data Availability

The original contributions presented in the study are included in the article/supplementary material, further inquiries can be directed to the corresponding author.
