# Corrigendum: Position statement and updated international guideline for safe and effective whole-body electromyostimulation training-the need for common sense in WB-EMS application

**DOI:** 10.3389/fphys.2023.1207584

**Published:** 2023-05-09

**Authors:** Wolfgang Kemmler, Michael Fröhlich, Oliver Ludwig, Christoph Eifler, Simon Von Stengel, Sebastian Willert, Marc Teschler, Anja Weissenfels, Heinz Kleinöder, Florian Micke, Nicolas Wirtz, Christoph Zinner, Andre Filipovic, Bernd Wegener, Joshua Berger, Alexandre Evangelista, Stefano D’ottavio, Jaskanwal Deep Singh Sara, Amir Lerman, Unai A. Perez De Arrilucea Le Floc’h, Abraham Carle-Calo, Angel Guitierrez, Francisco J. Amaro-Gahete

**Affiliations:** ^1^ Institute of Radiology University Hospital Erlangen (UKER), Erlangen, Germany; ^2^ Department of Sports Science, Rheinland-Pfälzische Technische Universität Kaiserslautern-Landau, Kaiserslautern, Germany; ^3^ German University for Prevention and Health Management, Saarbrücken, Germany; ^4^ Department of Rehabilitation Sciences, University of Witten/Herdecke, Witten, North RhineWestphalia, Germany; ^5^ Institute of Sports Science, Friedrich-Alexander University of Erlangen-Nürnberg, Erlangen, Germany; ^6^ Institute of Training Science and Sport Informatics, German Sport University Cologne, Cologne, North RhineWestphalia, Germany; ^7^ Central Library for Sport Sciences, German Sport University Cologne, Cologne, Germany; ^8^ University of Applied Sciences for Police and Administration of Hesse, Wiesbaden, Germany; ^9^ Soccer Club Paderborn 07, Paderborn, Germany; ^10^ Musculoskeletal University Center, Ludwig-Maximilian-University of Munich, Munich, Germany; ^11^ Experimental Physiology and Biochemistry, Center for Physical Education and Sport, University Federal Do Espírito Santo, Vitória, Brazil; ^12^ Clinical Sciences and Translational Medicine Department, University of Rome, Rome, Italy; ^13^ Department of Cardiovascular Medicine, Mayo College of Medicine, Rochester, MN, United States; ^14^ Department of Physical Education and Sport, University of Granada, Granada, Spain; ^15^ Department of Physiology, University of Granada, Granada, Spain; ^16^ Centro De Investigación Biomédica en Red Fisiopatología De La Obesidad y Nutrición (CIBERobn), Instituto De Salud Carlos III, Madrid, Spain; ^17^ Instituto De Investigación Biosanitaria De Granada (ibs.GRANADA), Granada, Spain

**Keywords:** recommendations, international consensus, whole-body electromyostimulation, electrical muscle stimulation, safety

In the published article, the citation of Author Jaskanwal Deep Singh Sara was incorrectly written as “Singh Sara JD” instead of “Sara JDS.”

The authors apologize for this error and state that this does not change the scientific conclusions of the article in any way. The original article has been updated.

